# Urban Green Spaces Set the Stage for Rare Interspecific Allopreening Between Crested Caracara ( *Caracara plancus* ) and Black Vultures ( *Coragyps atratus* )

**DOI:** 10.1002/ece3.72669

**Published:** 2026-01-12

**Authors:** Lori Boies, Franchesca Tinacba, Terry J. Shackleford

**Affiliations:** ^1^ St. Mary's University San Antonio Texas USA

**Keywords:** behavioral ecology, black vulture, crested caracara, interspecific allopreening, social grooming, urban ecology

## Abstract

Urban green spaces and wildlife corridors provide unique opportunities to observe how animals adapt and interact in human‐dominated landscapes. In San Antonio, Texas, both adjacent to and within the Phil Hardberger Park and the Robert L.B. Tobin Land Bridge, the largest wildlife bridge in the United States at the time of its construction, we documented a rare case of interspecific allopreening. On May 15, 2022, two Crested Caracaras ( *Caracara plancus* ) engaged in mutual head and neck allopreening, followed by two Black Vultures ( *Coragyps atratus* ) initiating preening of the Crested Caracara. Subsequent behaviors consisted of perching, self‐preening, and wing‐spreading by vultures, with no additional allopreening observed. The same behavior was also observed on February 4, 2023 within Phil Hardberger Park. While allopreening is well documented within species for social and hygienic purposes, interspecific allopreening is rare and has only been reported a few times globally and only in rural settings. Our observation is the first record of its kind in an urban green space and expands the ecological and geographic scope of such behavior, demonstrating that urban green infrastructure may act as a stage for uncommon avian social interactions. This record contributes to the growing body of urban wildlife research and highlights the value of systematic observation in cities for revealing overlooked aspects of avian behaviors.

## Graphical abstract

Observations in San Antonio, Texas documented rare interspecific allopreening between crested caracaras and black vultures at Phil Hardberger Park. This represents the first recorded instance of such behavior in an urban green space, expanding the known ecological and geographic scope of interspecies social interactions. The finding underscores the value of urban wildlife corridors for revealing overlooked avian behaviors and contributing to urban ecology research.

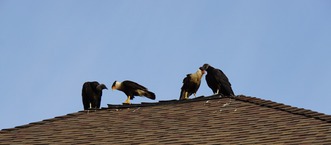

## 1 Introduction

Allopreening, or social grooming between individuals, is a widespread behavior among birds that typically occurs within species and fulfills both hygienic and social functions. It is most commonly associated with pair bonding, kinship maintenance, and ectoparasite control (Bush and Clayton 2018; Radford and Du Plessis 2006). Preening itself refers to the act of feather maintenance, whereby birds use their beaks to draw along each feather from base to tip, removing excess oil, debris, and parasites (Bush et al. 2023; Mya 2013). The act of preening is vital to avian survival because it ensures the integrity of plumage, which directly affects flight performance. Without regular preening, feathers may accumulate oil, debris, and parasites, which can substantially impair aerodynamic efficiency (Moreno‐Rueda 2017). In addition to cleaning, preening also distributes secretions from the uropygial gland, a specialized structure located at the base of the tail in many bird species. When compressed, this gland releases oil that birds spread across their feathers during preening. This oil contributes to feather health by enhancing flexibility and reducing brittleness, as well as suppressing bacterial growth on feathers (Alt et al. 2020; Braun et al. 2018; Moreno‐Rueda 2017).

While preening is a universal behavior among all bird species, there has been a more specific characteristic observed between birds within a flock. This observation is known as allopreening; the act in which birds, oftentimes within social groups, clean each other's feathers. Researchers hypothesize that allopreening is used to reinforce relationships between animals, allowing them to cooperate more positively (Jensen et al. 2023). In most flocks, allopreening occurs intraspecifically (between members of the same species). However, recent observations have documented instances of interspecific allopreening, in which individuals of different species engage in this behavior (Zhou and Zhang 2022). Reports of interspecific allopreening between Crested Caracaras ( *Caracara plancus* ) and Black Vultures ( *Coragyps atratus* ) exist in the literature; however, these accounts are limited to rural settings and describe only isolated, individual interactions (Ng and Jasperson 1984; Souto et al. 2009). Such interactions are comparatively rare and have therefore become a focal point of ornithological research. The unusual nature of interspecific allopreening has prompted longstanding questions regarding its function and evolutionary significance.

Urban environments add another important dimension to this natural observation. As cities expand, they increasingly host dense populations of adaptable species, reshaping avian communities and altering the frequency and context of interspecific encounters (Shochat et al. 2006). While urban habitats often restrict biodiversity, they can also create novel opportunities for wildlife interactions by concentrating resources and species in shared spaces. San Antonio, Texas, provides an especially valuable example of this with the extensive Phil Hardberger Park along with the Robert L.B. Tobin Land Bridge, which connects both sides of the park above a six‐lane parkway (Kirkpatrick 2020). This land bridge was the largest wildlife crossing in the United States at the time of its construction and until the Annenberg Wildlife Crossing in Agoura Hills, California opens in 2025 (California 2025). The Robert L.B. Tobin Land Bridge in San Antonio reconnects fragmented habitat across a major roadway, enabling safe movement of wildlife and enhancing ecological connectivity within the city. Early reports from motion‐triggered cameras have shown evidence that there are at least 16 species of wildlife that have utilized the bridge so far during a 5‐year study, which is ongoing (Protozanova 2021; Rodriguez 2021; Torres 2023). Such corridors may not only preserve biodiversity but also provide areas for observing rare or understudied behaviors.

Our record of interspecific allopreening between a Black Vulture and a Crested Caracara in an urban San Antonio green space highlights how rare affiliative behaviors may emerge at the intersection of species ecology and human‐modified landscapes, emphasizing the value of systematic observation in uncovering underappreciated dimensions of avian sociality.

## 2 Field Observations

**FIGURE 1:**
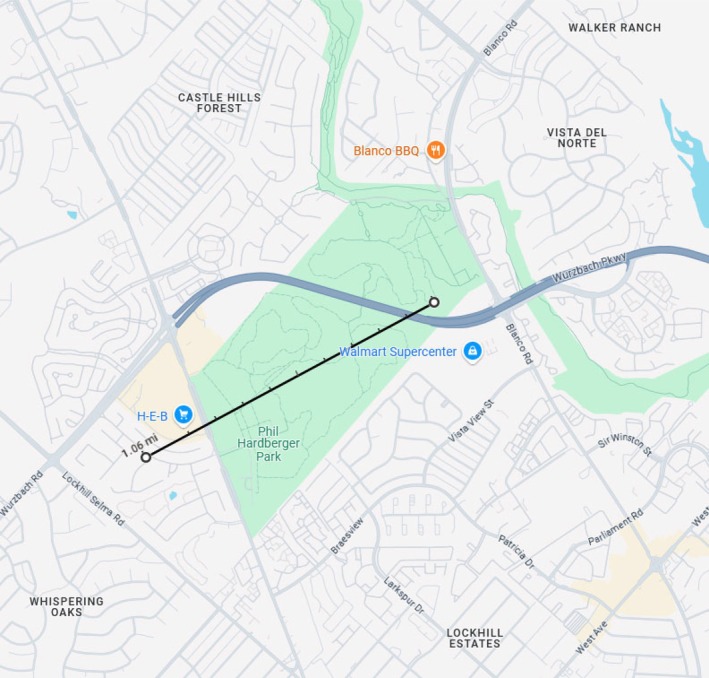
Locations of the two observations in San Antonio, Texas. Map adapted from Google Maps (Google, accessed April 1, 2025). John Wiley & Sons Ltd. remains neutral with regard to jurisdictional claims in published maps (Google Maps, n.d.).

Two distinct occurrences of interspecific allopreening between Black Vultures and Crested Caracara were documented by *ad libitum* sampling, in which notable behaviors are recorded opportunistically when observed (Altmann 1974). The observations occurred within a 10 month period and within approximately one mile of each other (Figure 1). The locations of these observations both occurred in San Antonio, Texas, United States, but one was in an urban neighborhood on a residential roof and the other within a 330‐acre sustainable natural urban park.

**FIGURE 2:**
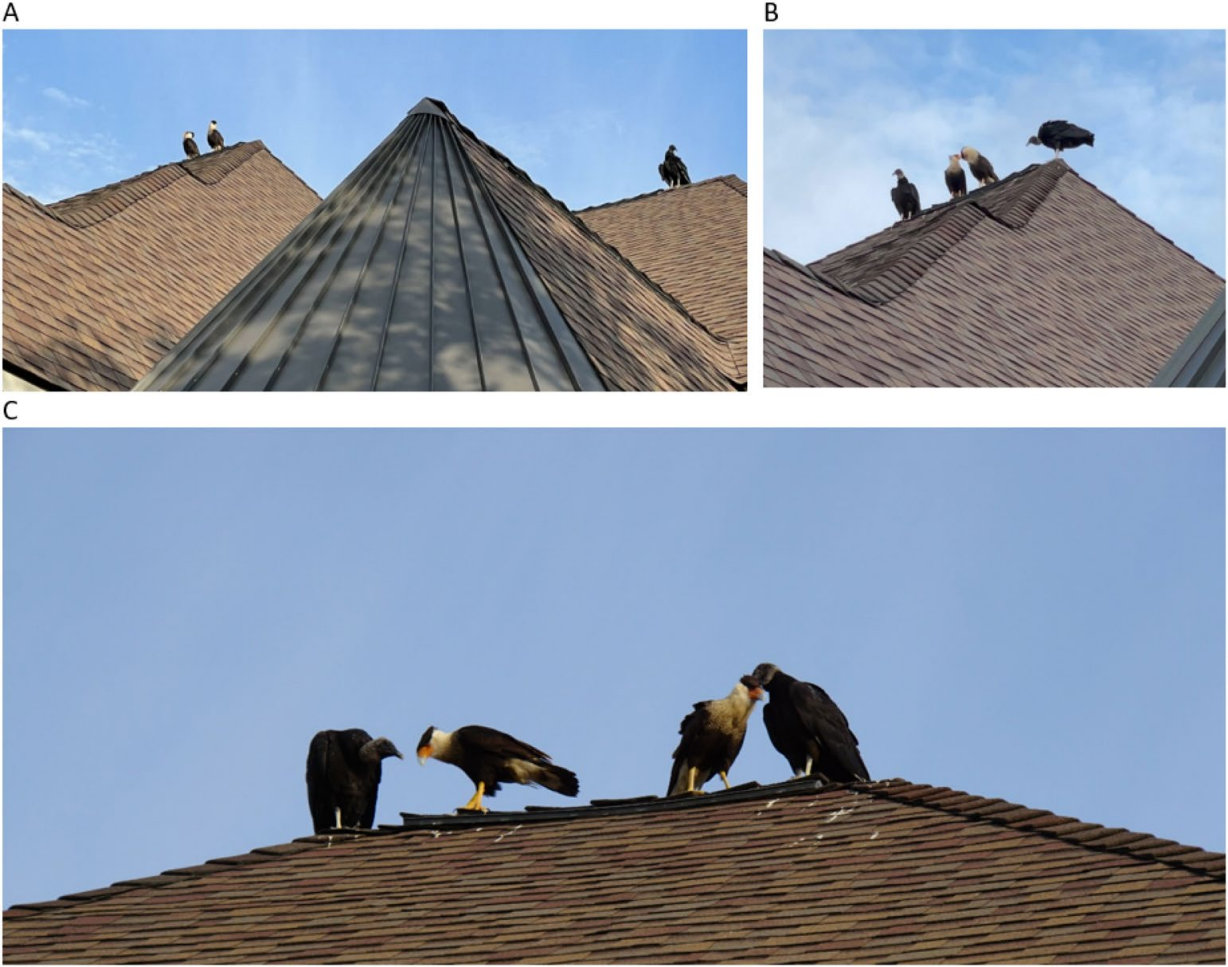
Interaction of a pair of crested caracara with black vultures within an urban environment. (A) Pair of crested caracaras perched on a residential roof with a black vulture in the vicinity. (B) Pair of crested caracaras participating in intraspecific allopreening with black vultures in the vicinity. (C) Pairs of black vultures and crested caracaras participating in rarely observed interspecific allopreening.

On the morning of May 18, 2022, Crested Caracara were first observed as a pair on the roof of a two‐story residential home in the 12,000 block of Indigo Bend in San Antonio, Texas 78,230 (29° 33′ 5.3856″ N and 98° 32′ 11.328″ W) with a single black vulture also perched on a different area of the roof (Figure 2A). The neighborhood of the residence had a mixed development of homes and narrow greenspaces that were left untouched to remain natural (behind the residential home of observation was a greenspace of approximately 30.48 m (100 ft) wide by 312.42 m (1025 ft) long). In relation to the topography of the land and the architectural design of the home, this is the highest perch point in the neighborhood. Various bird species commonly perched on this roof to include Crested Caracara ( *Caracara plancus* ), Black Vulture ( *Coragyps atratus* ), Cooper's Hawk ( *Accipiter cooperii* ), and the Great Horned Owl ( *Bubo virginianus* ).

The pair of Crested Caracara were initially participating in intraspecific allopreening with a head down display, once this activity commenced, a Black Vulture on the other part of the roof joined the immediate vicinity of the Crested Caracara and another Black Vulture also joined within 5 min of the observed activity (Figure 2B). The Crested Caracara then assumed a head‐down display thus inviting the Black Vulture pair to commence interspecific allopreening (Figure 2C). The Crested Caracaras were both preened on the top of the head, nape of the neck, and breast. The Crested Caracara and black vultures did not switch the preening pairs and engaged in this behavior for approximately 20 min with breaks in which the birds perched next to each other without moving away as summarized in Table 1. The observation ended with the Black Vultures disengaging and either leaving the vicinity or moving to another part of the roof (see Appendix S1).

**TABLE 1:** Chronological summary of allopreening and associated behaviors between Crested Caracaras ( *Caracara plancus* ) and Black Vultures ( *Coragyps atratus* ) observed in San Antonio, Texas, on 15 May 2022.

Time	Preening duration (seconds)	Initiator	Recipient	Interaction type	Body area preened (Head/Neck, wing)	Notes
07:05 am	96	Crested Caracara	Crested Caracara	IntraAllopreening	Head/Neck	Alternating roles with one Black Vulture nearby
07:07 am	33	Black Vulture	Crested Caracara	Interspecific Allopreening	Head/Neck	Vulture initiated
07:10 am	78	Black Vulture	__	Perching, self‐preening	__	Multiple vultures present, no allopreening
07:12 am	242	Black Vulture	__	Perching	__	Continued passive presence, no allopreening, wing‐spreading, solitary behaviors.

*Note:* Events occurred between 07:05 and 07:21 am under partly cloudy conditions, with a temperature of 21.7°C (71.1°F), wind speed of 9.25kph (5.75 mph), and no precipitation.

**FIGURE 3:**
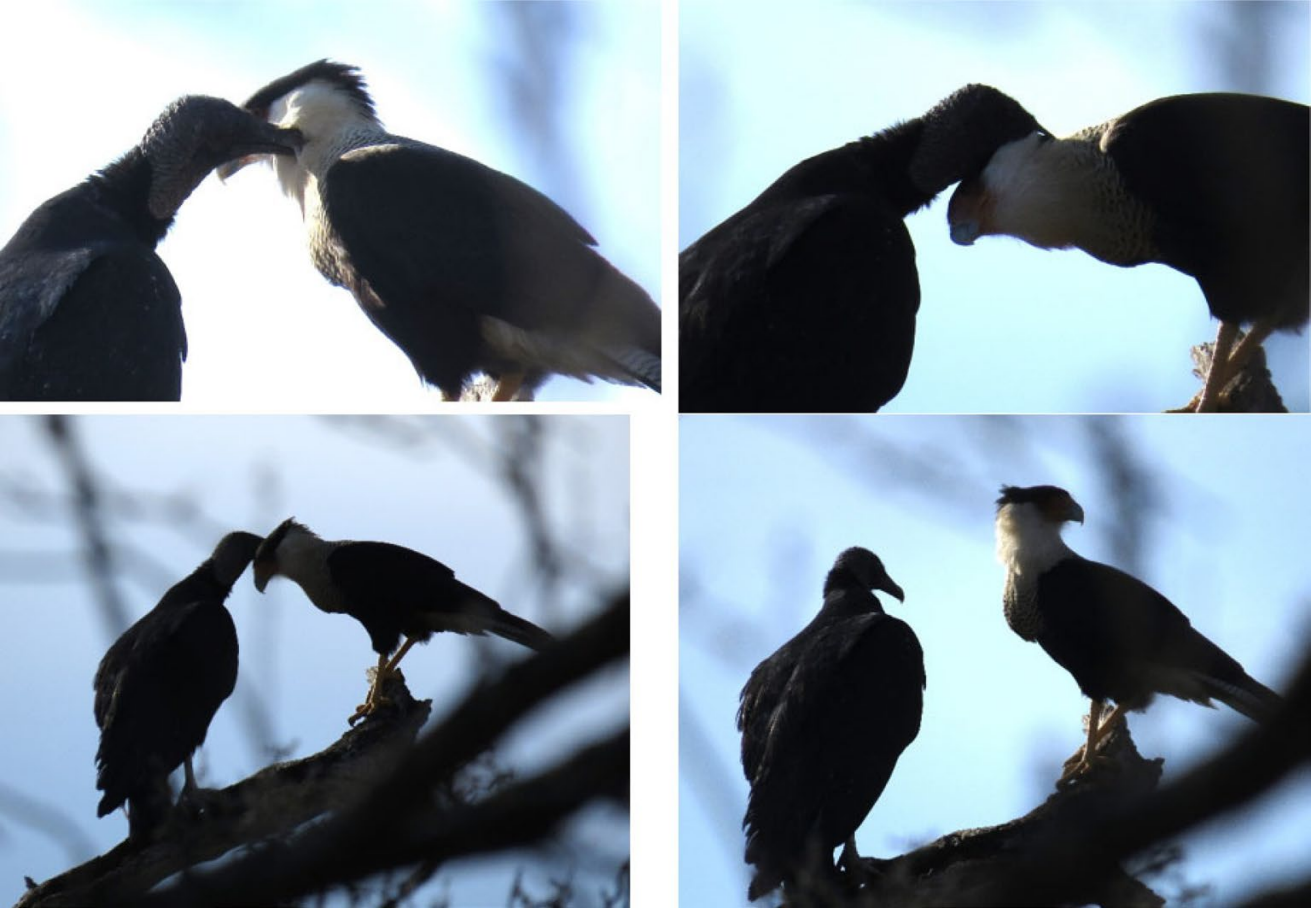
Interspecific allopreening session between black vulture and crested caracara in Phil Hardberger natural urban park. Photographs by Lora Reynolds.

Within the same geographic region, this behavior was also observed the next year on February 4, 2023 when a single Crested Caracara and Black Vulture were observed in Phil Herberger Park on the Water Loop Trail (29° 33′ 31.2372″ N and 98° 31′ 16.122″ W) (Reynolds 2023). The birds involved in the interspecific allopreening were within a wooded part of the park and interacting on a tree branch. The Crested Caracara did present itself with a lowered‐head preening invitation (Figure 3). Preening in this instance was isolated to the head and neck regions of the Crested Caracara and the birds also spent time apart on the tree limb. For both observations, the Crested Caracara presented itself through a lowered‐head preening invitation and all birds involved interacted in a cooperative manner to enable the behavior.

**FIGURE 4:**
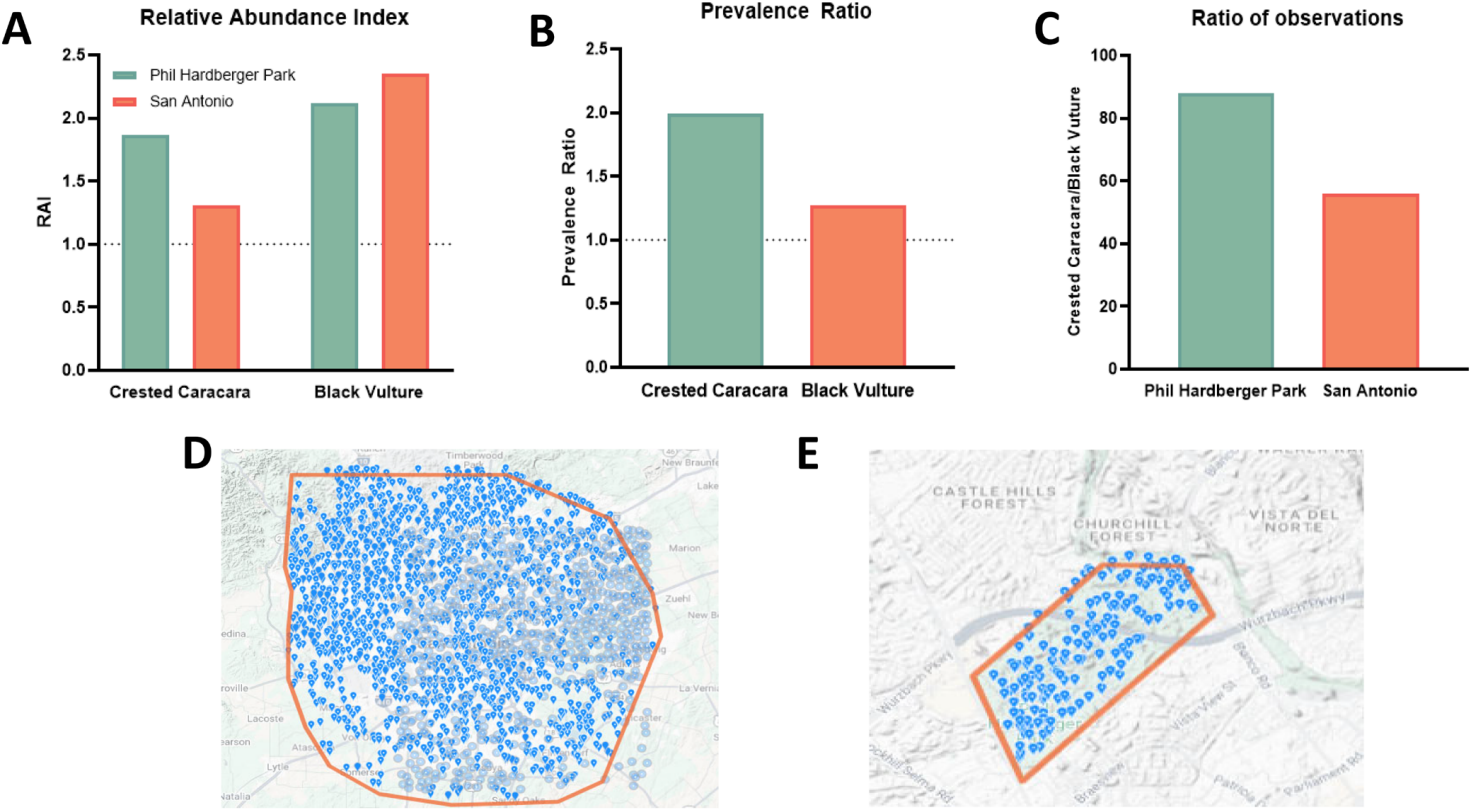
Observations of Crested Caracara and Black Vulture show higher abundance in Phil Hardberger Park compared to San Antonio citywide. (A) Relative abundance index for both species in Phil Hardberger Park and across San Antonio (RAI = observations of species/total bird observations × 100). (B) Prevalance ratio of observations in Phil Hardberger Park compared to citywide prevalence ((observations in park/observations in city) × 100; values > 1 indicate higher prevalence in the park). (C) Ratio of Caracara to Black Vultures in Phil Hardberger Park compared to San Antonio. (D) Map of bird species observations across the greater San Antonio area from iNaturalist. (E) Map of bird species observations within Phil Hardberger Park from iNaturalist (iNaturalist Research‐Grade Observations Dataset, n.d.).

Observations from iNaturalist records between January 1, 2014 and September 1, 2025 documented the relative prevalence of Crested Caracara and Black Vulture in San Antonio and within Phil Hardberger Park (iNaturalist Research‐Grade Observations Dataset, n.d.). This represents the complete publicly available dataset for Crested Caracaras and Black Vultures available at the time of our analysis and allows for the ability to establish a pattern of species observations over time with an adequate sample size for both species. A total of 84,073 birds were observed in the San Antonio area while 1179 were observed in Phil Hardberger Park. A total of 22 Crested Caracara and 25 Black Vulture observations were recorded in the park, compared to 1103 Crested Caracara and 1972 Black Vulture observations in the city of San Antonio (Figure 4). Mapping records of regions defined in iNaturalist show that the observations of the ratio and relative abundance of these two species is unique in Phil Hardberger Park when compared to those seen in the city of San Antonio. When standardized as a relative abundance index (RAI = [observations of species/total bird observations] × 100), Phil Hardberger Park compared to San Antonio (Figure 4A). Prevalence ratios quantified how frequently each species was recorded in Phil Hardberger Park relative to San Antonio as a whole (Prevelence = [observations in park/observations in city] × 100). When compared directly, the Crested Caracaras were 1.99 times more prevalent in the park than citywide, while Black Vultures were 1.27 times more prevalent than citywide, representing an overall 57% increase of Crested Caracara in the park (Figure 4B). The ratio of Crested Caracara to Black Vulture was higher in Phil Hardberger Park compared to San Antonio 88% versus 56% (Figure 4C). These findings indicate that Phil Hardberger Park functions as a local hotspot for large scavenging birds, with higher‐than‐expected densities relative to the broader urban region. The enhanced availability of natural resources, combined with the continuity provided by the Robert L.B. Tobin Land Bridge, may promote increased overlap and interaction between species. This concentration of birds in shared urban habitats may provide the ecological context in which rare interspecific interactions, such as the allopreening events documented here, are more likely to be observed.

## 3 Discussion

Allopreening is widespread among birds and is typically associated with pair bonding and ectoparasite control. The functional and sociobiological purpose of interspecific allopreening between birds has not yet been fully elucidated. The urban context of these observations is unique as other documented instances of Black Vultures and Crested Caracara participating in cross‐species allopreening have occurred in rural, natural areas or within the periphery of urban areas (Ng and Jasperson 1984; Souto et al. 2009). These observations took place in the midst of a busy, urban city. The area between the neighborhood and the urban park includes a busy parkway that bisects the park (the landbridge helps to facilitate animal movement), other neighborhoods, and a large shopping center to include restaurants, an emergency room, retail shopping, and a grocery store. Previous reports have indicated that Crested Caracara and Black Vultures will interact around feeding sites with the Crested Caracara assuming a more dominant role within this interaction (Buckley 2020). Additionally, this is the first report in the literature of two pairs of birds from different species allopreening in close proximity. This underlies questions regarding the sociobiological mechanisms behind this behavior.

While the functional significance of interspecific allopreening remains unclear, it is known to play an important role in ectoparasite removal. Vultures are scavenging animals that are exposed to parasites and pathogens and exhibit specialized morphological and microbial adaptations that reduce their susceptibility to infections. Their relatively bare head and neck are more resistant to lice, mites, and other parasites as the exposed skin is easier to keep clean and is also exposed to solar radiation and desiccation. Vultures host a specialized skin microbiota that produces antimicrobial compounds that can reduce ectoparasitic organisms (Lobello et al. 2025). Together, this suggests that Black Vultures have a reduced need for ectoparasite removal through allopreening. In contrast, Crested Caracaras have fully feathered heads and necks, making them more vulnerable to ectoparasites and reliant on grooming behaviors like allopreening for parasite control. These feathered regions are also anatomically difficult for birds to access during self‐preening, which may increase the importance of allopreening as a hygienic behavior. The contrast between these species may explain why interspecific allopreening was observed, where a Black Vulture preened a Crested Caracara and could point to a mutualistic or facilitative behavior between the species. Further studies are needed to determine the validity of the relationship between the species.

The observations presented here took place in an urban environment that was purposefully planned to include green spaces. The neighborhood of observations was located near Phil Hardberger Park and its land bridge, and its design intentionally preserved natural greenbelts behind the homes. These undeveloped areas varied from approximately 7.20 m (25 ft) to 83.82 m (275 ft) in width, with the longest continuous length extending 312.42 m (1025 ft). Other observations were described within the 330‐acre sustainable natural urban park. Large green spaces within metropolitan settings can concentrate avian populations at higher densities than are typically observed in rural landscapes. Of particular note, the land bridge was the largest wildlife crossing of its kind in the United States when completed in 2020. This structure not only provides safe passage for both humans and wildlife but also enhances opportunities for interspecific interactions in an urban context by facilitating movement across fragmented habitats. Literature supports the idea that large urban parks may facilitate Crested Caracara presence by mimicking the open, heterogenous landscapes these urban‐adaptors may preferentially use. Habitat modeling in Texas shows that Crested Caracara occurrence increases in semi‐open landscapes with edge habitats, like those commonly seen in urban parks (Smith et al. 2017). Additionally, parks may provide predictable access to scavenging opportunities (trash, carrion associated with nearby roads, and concentrations of prey species) which align with Crested Caracara's opportunistic foraging strategies. Recent long‐term survey analyses have provided evidence of Crested Caracara expanding their range northward in Texas, and increasingly utilizing landscapes altered by human activity such as parks, golf courses, utility corridors, and other greenspaces (Smith and Dwyer 2024). These large, open greenspaces may imitate native breeding habitats like grasslands, farmlands, brushland/open fields where they will nest in trees, shrubs, and sometimes human‐modified structures (CRESTED CARACARA|The Texas Breeding Bird Atlas, n.d.). The literature also emphasizes avian species richness increases in urban areas where there are large heterogenous patches of natural vegetation maintained (Chiron et al. 2024; Sandström et al. 2006). Collectively, these studies suggest that large urban parks and other human‐modified greenspaces can act as habitat nodes or movement corridors, supporting Crested Caracara presence rather than excluding them.

Vultures are known to contribute to ecosystem homeostasis through their ability to dispose of carcasses and contribute to hygiene in the ecosystem. They may play another role in hygienic aid by removing ectoparasites from a body area on the Crested Caracara that the Black Vultures themselves are free from maintaining due to their unique morphological and microbial adaptations. Urban growth has been shown to lead to declined populations through loss of the species' nesting habitat (Morrison and Dwyer 2023). However, urban areas can support elevated densities of adaptable species which can lead to greater spatial overlap and interaction opportunities than in rural habitats. While rural Crested Caracara and Black Vultures may share roosts and foraging grounds, the forced proximity of urban greenbelts and perching sites (e.g., rooftops, towers, and bridges) can amplify opportunities for rare affiliative behaviors such as interspecific allopreening.

Our iNaturalist analysis revealed clear gradients in the relative abundance of Crested Caracaras and Black Vultures across spatial scales, with the highest relative values in Phil Hardberger Park and lower indices across San Antonio. The elevated prevalence of Crested Caracara and Black Vultures within Phil Hardberger Park as seen in Figure 4 highlights the importance of urban green spaces and wildlife corridors in shaping species distributions. These species are often underrepresented in urban monitoring due to their wide‐ranging movements, yet our analysis of iNaturalist community scientist data indicates that Phil Hardberger Park may provide concentrated foraging and roosting opportunities that attract scavengers in that area compared to the surrounding city (iNaturalist Research‐Grade Observations Dataset, n.d.). This data supports broader urban ecology findings that parks and habitat connectors function as biodiversity reservoirs. Crested Caracara are urban‐adaptors and do not thrive in dense city interiors, and their best outcomes may be associated with open landscapes that are adjacent to human influence, such as Phil Hardberger Park, rather than in heavy built‐up urban cores. Notably, the higher relative abundance of Black Vultures in the park may increase the likelihood of interactions with other scavengers such as Crested Caracaras, allowing for unusual behaviors like the interspecific allopreening documented in this study. By combining behavioral observations with citizen science data, we show how urban ecology research can link community‐level prevalence patterns with rare, fine‐scale social behaviors.

Our observation of interspecific allopreening provides a documented case of this rare behavior that adds to only a few documented cases previously observed in rare areas of Texas and South/Central America (Birds of the World, n.d.; Buckley 2020; da Silva and Santos do Carmo 2015; Souto et al. 2009; Palmeira 2008; Ng and Jasperson 1984). These observations support the emerging view that interspecific allopreening may play a role in parasite management and possibly other roles between the species. Unlike previous reports, our observation occurred in the middle of a major U.S. city in an urban green space both adjacent to and within the Phil Hardberger Park and the Robert L.B. Tobin Land Bridge, demonstrating how landscapes can provide opportunities for these rare interactions. This study is the first report of Black Vulture–Crested Caracara allopreening in an urban setting, expanding both the ecological scope and the geographic distribution of this uncommon behavior. This supports the theory that human‐modified landscapes can promote wildlife interactions and reveal behavior rarely seen in rural habitats.

## Author Contributions

**Lori Boies:** conceptualization (lead), data curation (equal), formal analysis (lead), investigation (lead), methodology (equal), project administration (lead), writing – original draft (equal), writing – review and editing (lead). **Franchesca Tinacba:** conceptualization (supporting), investigation (supporting), methodology (equal), writing – original draft (equal), writing – review and editing (supporting). **Terry J. Shackleford:** conceptualization (lead), data curation (equal), formal analysis (equal), investigation (supporting), methodology (equal), project administration (equal), software (equal), writing – original draft (equal), writing – review and editing (lead).

## Funding

The authors have nothing to report.

## Conflicts of Interest

The authors declare no conflicts of interest.

## Supporting information


**Appendix S1:** Sequence of rooftop interaction among Crested Caracaras and Black Vultures documented on May 15, 2022. (A) Mutual head/neck allopreening between two Caracaras. (B) Interspecific allopreening initiated by a Black Vulture directed toward a Caracara (C) Subsequent perching, self‐preening, and wing‐spreading behaviors by Black Vultures. Images are labeled by camera file labeling of serial photos.

## Data Availability

Data sharing not applicable to this article as no datasets were generated or analyzed during the current study.
