# Supplementary material for: Urban Green Spaces Set the Stage for Rare Interspecific Allopreening Between Crested Caracara ( Caracara plancus ) and Black Vultures ( Coragyps atratus )
Source: Ecol Evol. 2026 Jan 12;16(1):e72669. doi: 10.1002/ece3.72669 (PMC12794025; doi:10.1002/ece3.72669)
Supplement: Supplementary file 1 — Appendix S1: Sequence of rooftop interaction among Crested Caracaras and Black Vultures documented on May 15, 2022. (A) Mutual head/neck allopreening between two Caracaras. (B) Interspecific allopreening initiated by a Black Vulture directed toward a Caracara (C) Subsequent perching, self‐preening, and wing‐spreading behaviors by Black Vultures. Images are labeled by camera file labeling of serial photos. [file ECE3-16-e72669-s001.docx]

Appendix 1: Sequence of rooftop interaction among Crested Caracaras and Black Vultures documented on May 15, 2022. A) Mutual head/neck allopreening between two Caracaras. B) Interspecific allopreening initiated by a Black Vulture directed toward a Caracara C) Subsequent perching, self-preening, and wing-spreading behaviors by Black Vultures. Images are labeled by camera file labeling of serial photos.

1. DSC00330 – DSC00342

| 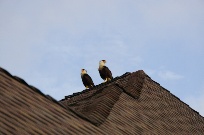 | 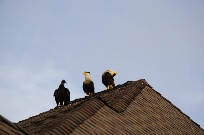 | 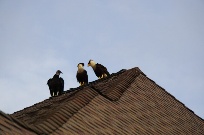 | 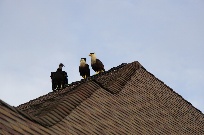 | 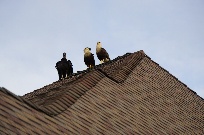 | 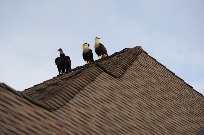 |
| --- | --- | --- | --- | --- | --- |
| 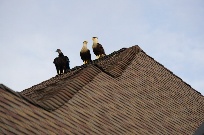 | 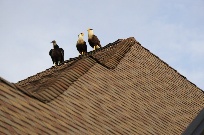 | 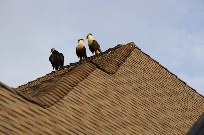 | 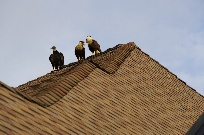 | 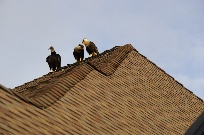 | 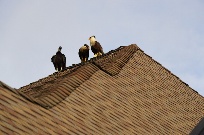 |

B. DSC00343 – DSC00348

| 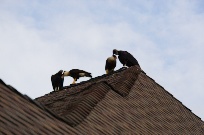 | 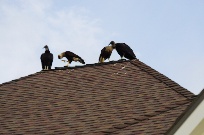 | 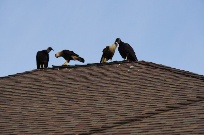 | 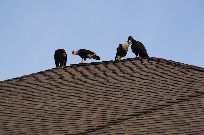 | 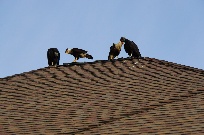 | 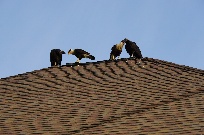 |
| --- | --- | --- | --- | --- | --- |

C. DSC00351-DSC00354

| 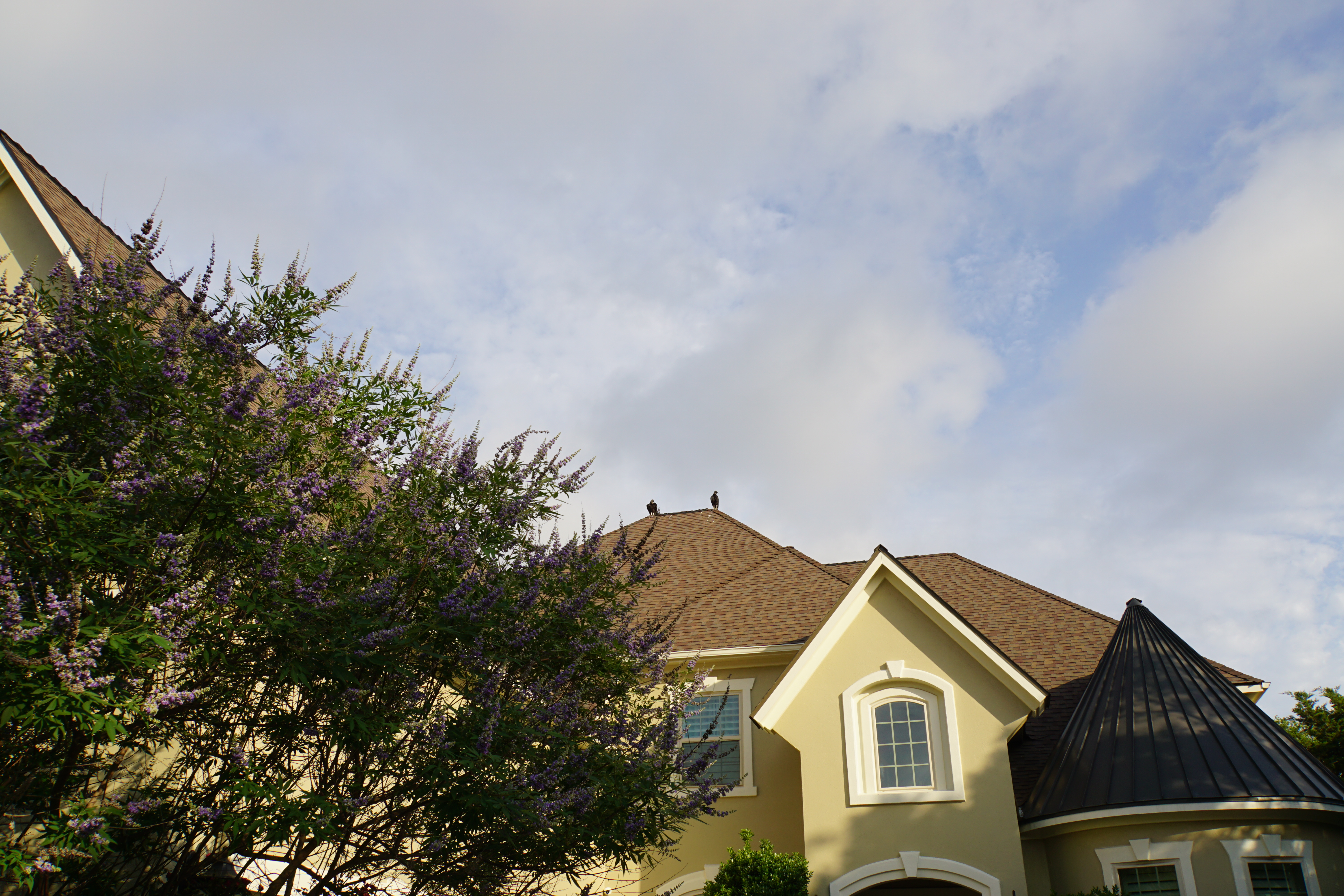 | 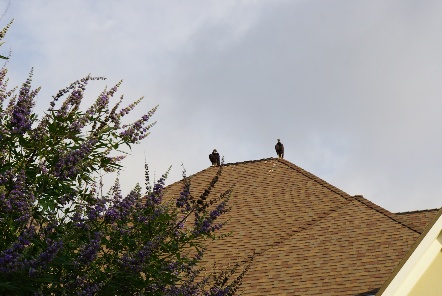 | 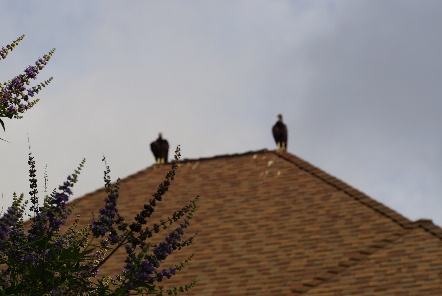 |
| --- | --- | --- |

D. DSC00361 – DSC00402

| 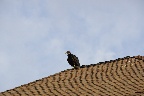 | 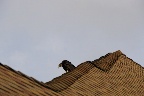 | 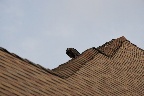 | 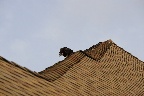 | 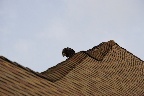 | 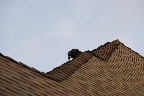 | 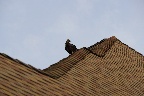 | 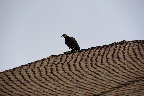 |
| --- | --- | --- | --- | --- | --- | --- | --- |
| 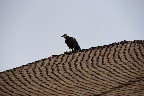 | 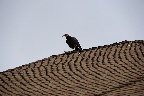 | 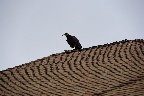 | 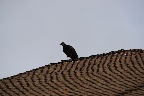 | 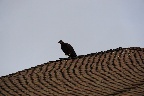 | 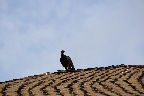 | 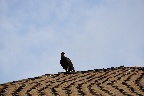 | 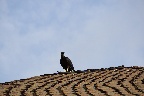 |
| 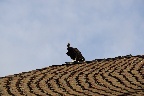 | 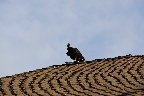 | 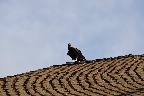 |  |  |  |  |  |
